# 2,9-Dimethyl-1,10-phenanthrolin-1-ium tetra­chloridoferrate(III) methanol monosolvate

**DOI:** 10.1107/S1600536812025111

**Published:** 2012-06-13

**Authors:** Ehsan Bahojb Noruzi, Nasser Safari, Vahid Amani, Behrouz Notash

**Affiliations:** a Department of Chemistry, Shahid Beheshti University, G. C., Evin, Tehran 1983963113, Iran

## Abstract

In the title compound, (C_14_H_13_N_2_)[FeCl_4_]·CH_3_OH, the 2,9-dimethyl-1,10-phenanthrolin-1-ium cation, FeCl_4_^−^ anion and methanol solvent mol­ecule lie on a twofold rotation axis. Due to symmetry, the H atom on the N atom of the cation is half-occupied. In the anion, the Fe^III^ atom has a tetra­hedral geometry. H atoms of the methanol mol­ecule are disordered over two sets of sites around the twofold axis. In the crystal, π–π contacts between the pyridine rings and between the pyridine and benzene rings [centroid–centroid distances = 3.6535 (16) and 3.5522 (17) Å] and inter­molecular O—H⋯N and N—H⋯O hydrogen bonds stabilize the structure.

## Related literature

For related structures, see: Abboud *et al.* (2005 ▶); Amani *et al.* (2007 ▶, 2009 ▶); Khavasi *et al.* (2008 ▶); Moreno *et al.* (2006 ▶); Morsali (2005 ▶); Veidis *et al.* (1981 ▶); Yousefi *et al.* (2007 ▶); Yu *et al.* (2006 ▶).
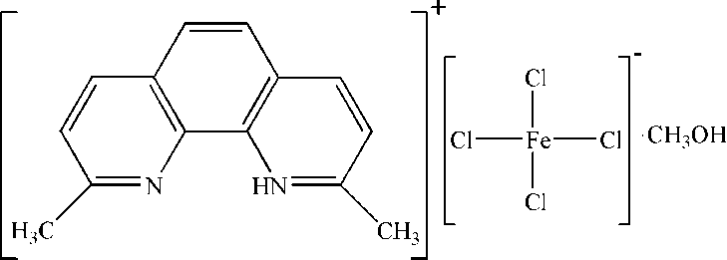


## Experimental

### 

#### Crystal data

(C_14_H_13_N_2_)[FeCl_4_]·CH_4_O
*M* *_r_* = 438.96
Monoclinic, 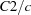

*a* = 13.067 (3) Å
*b* = 20.377 (4) Å
*c* = 7.2810 (15) Å
β = 109.03 (3)°
*V* = 1832.7 (7) Å^3^
*Z* = 4
Mo *K*α radiation
μ = 1.41 mm^−1^
*T* = 120 K
0.20 × 0.15 × 0.10 mm

#### Data collection

Stoe IPDS-2T diffractometer
Absorption correction: numerical (*X-SHAPE* and *X-RED*; Stoe & Cie, 2002 ▶) *T*_min_ = 0.766, *T*_max_ = 0.872
9967 measured reflections
2464 independent reflections
2075 reflections with *I* > 2σ(*I*)
*R*_int_ = 0.049

#### Refinement

*R*[*F* ^2^ > 2σ(*F* ^2^)] = 0.045
*wR*(*F* ^2^) = 0.099
*S* = 1.10
2464 reflections
112 parameters
H atoms treated by a mixture of independent and constrained refinement
Δρ_max_ = 0.84 e Å^−3^
Δρ_min_ = −0.84 e Å^−3^

### 

Data collection: *X-AREA* (Stoe & Cie, 2002 ▶); cell refinement: *X-AREA*; data reduction: *X-RED* (Stoe & Cie, 2002 ▶); program(s) used to solve structure: *SHELXS97* (Sheldrick, 2008 ▶); program(s) used to refine structure: *SHELXL97* (Sheldrick, 2008 ▶); molecular graphics: *ORTEP-3* (Farrugia, 1997 ▶); software used to prepare material for publication: *WinGX* (Farrugia, 1999 ▶).

## Supplementary Material

Crystal structure: contains datablock(s) I, global. DOI: 10.1107/S1600536812025111/hy2554sup1.cif

Structure factors: contains datablock(s) I. DOI: 10.1107/S1600536812025111/hy2554Isup2.hkl

Additional supplementary materials:  crystallographic information; 3D view; checkCIF report

## Figures and Tables

**Table 1:** Hydrogen-bond geometry (Å, °)

*D*—H⋯*A*	*D*—H	H⋯*A*	*D*⋯*A*	*D*—H⋯*A*
O1—H1⋯N1	0.84	2.33	2.751 (4)	112
N1—H1*D*⋯O1	0.65 (12)	2.10 (10)	2.751 (4)	175 (14)

**Fig. 1.**
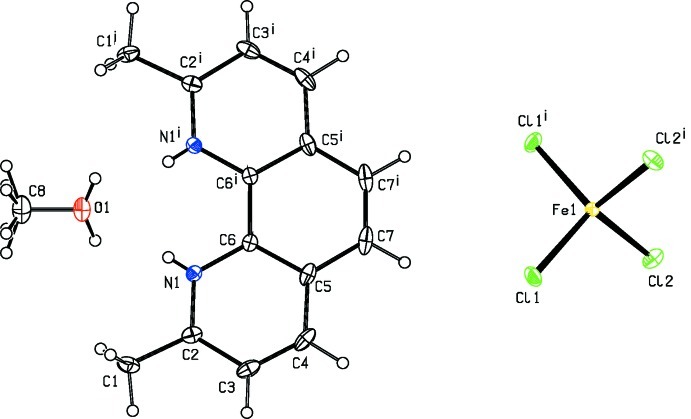
The molecular structure of the title compound. Displacement ellipsoids are drawn at the 50% probability level. [Symmetry code: (i) 2-x, y, 1/2-z.]

**Fig. 2.**
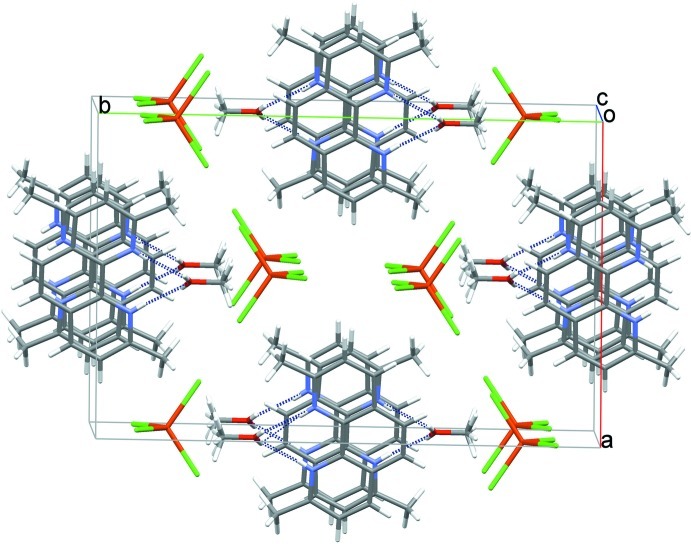
Crystal packing diagram for the title compound. Hydrogen bonds are shown as dashed lines.

## supplementary crystallographic information

### Comment

In the recent years, we reported the synthesis and crystal structures of iron(III) proton transfer complexes, such as [Fe(bipy)Cl_4_][bipy.H] (Amani *et al.*, 2007), [Fe(phen)Cl_4_][phen.H] (Khavasi *et al.*, 2008), [Fe(4,4'-dmbpy)Cl_4_][4,4'-dmbpy.H] (Amani *et al.*, 2009) (bipy = 2,2'-bipyridine, phen = 1,10-phenanthroline, 4,4'-dmbpy = 4,4'-dimethyl-2,2'-bipyridine). Several proton transfer systems using 2,9-dimethyl-1,10-phenanthroline as proton donor molecules, such as [Me_2_phen.H](ClO_4_) (Morsali, 2005), [Me_2_phen.H](NO_3_) (Yu *et al.*, 2006), [Me_2_phen.H][Ru(CO)_3_Cl_3_] (Moreno *et al.*, 2006) and [Me_2_phen.H]_2_[PtCl_6_] (Yousefi *et al.*, 2007) (Me_2_phen.H = 2,9-dimethyl-1,10-phenanthrolinium) have been synthesized and characterized by single-crystal X-ray diffraction methods. Also, the structure of [Me_2_phen.H][FeCl_4_] was reported (Veidis *et al.*, 1981). We report herein the synthesis and crystal structure of the title compound.

The molecular structure of the title compound is shown in Fig. 1. The asymmetric unit of the title compound contains half of a protonated 2,9-dimethyl-1,10-phenanthrolinium cation, half of a FeCl_4_^-^ anion and half of a methanol solvent molecule. In the anion, the Fe^III^ atom has a tetrahedral coordination. The Fe—Cl bond lengths and angles are within normal range (Abboud *et al.*, 2005; Amani *et al.*, 2007). In the crystal, intermolecular O—H···N and N—H···O hydrogen bonds (Table 1) and π–π interactions between the pyridine and benzene rings, *Cg*1···*Cg*1^i^ = 3.6535 (16) and *Cg*1···*Cg*2^ii^ = 3.5522 (17) Å [*Cg*1 and *Cg*2 are the centroids of the N1/C2–C6 ring and the C5, C6, C7, C5^iii^, C6^iii^, C7^iii^ ring; symmetry codes: (i) *x*, 1-*y*, -1/2+*z*; (ii) 2-*x*, 1-*y*, -*z*; (iii) 2-*x*, *y*, 1/2-*z*], stabilize the structure (Fig. 2).

### Experimental

For the preparation of the title compound, a solution of 2,9-dimethyl-1,10-phenanthroline (0.25 g, 1.20 mmol) in acetonitrile (10 ml) was added to a solution of FeCl_3_.6H_2_O (0.11 g, 0.40 mmol) in methanol (10 ml) and the resulting yellow solution was stirred at 313 K for 2 h. This solution was left to evaporate slowly at room temperature. After 5 days, yellow needle crystals of the title compound were isolated (yield: 0.13 g, 74.0%; m. p. 426 K).

### Refinement

H atoms bonded to N atom was found in difference Fourier map and refined isotropically. H atoms bonded to C and O atoms were positioned geometrically and refined as riding atoms, with O—H = 0.84, C—H = 0.95 (aromatic) and 0.98 (methyl) Å and with *U*_iso_(H) = 1.2(1.5 for methyl and hydroxyl)*U*_eq_(C,O)). H atoms of the methanol solvent molecule are disordered over two sets of sites around a twofold axis.

### Crystal data

**Table:**

(C_14_H_13_N_2_)[FeCl_4_]·CH_4_O	*F*(000) = 892
*M**_r_* = 438.96	*D*_x_ = 1.591 Mg m^−^^3^
Monoclinic, *C*2/*c*	Mo *K*α radiation, λ = 0.71073 Å
Hall symbol: -C 2yc	Cell parameters from 2464 reflections
*a* = 13.067 (3) Å	θ = 3.1–29.2°
*b* = 20.377 (4) Å	µ = 1.41 mm^−^^1^
*c* = 7.2810 (15) Å	*T* = 120 K
β = 109.03 (3)°	Needle, yellow
*V* = 1832.7 (7) Å^3^	0.20 × 0.15 × 0.10 mm
*Z* = 4	

### Data collection

**Table:**

Stoe IPDS-2T diffractometer	2464 independent reflections
Radiation source: fine-focus sealed tube	2075 reflections with *I* > 2σ(*I*)
Graphite monochromator	*R*_int_ = 0.049
ω scans	θ_max_ = 29.2°, θ_min_ = 3.1°
Absorption correction: numerical (*X-SHAPE* and *X-RED*; Stoe & Cie, 2002)	*h* = −17→17
*T*_min_ = 0.766, *T*_max_ = 0.872	*k* = −27→27
9967 measured reflections	*l* = −9→9

### Refinement

**Table:**

Refinement on *F*^2^	Primary atom site location: structure-invariant direct methods
Least-squares matrix: full	Secondary atom site location: difference Fourier map
*R*[*F*^2^ > 2σ(*F*^2^)] = 0.045	Hydrogen site location: inferred from neighbouring sites
*wR*(*F*^2^) = 0.099	H atoms treated by a mixture of independent and constrained refinement
*S* = 1.10	*w* = 1/[σ^2^(*F*_o_^2^) + (0.0412*P*)^2^ + 3.5467*P*] where *P* = (*F*_o_^2^ + 2*F*_c_^2^)/3
2464 reflections	(Δ/σ)_max_ = 0.001
112 parameters	Δρ_max_ = 0.84 e Å^−^^3^
0 restraints	Δρ_min_ = −0.84 e Å^−^^3^

### Special details

**Table:**

Geometry. All e.s.d.'s (except the e.s.d. in the dihedral angle between two l.s. planes) are estimated using the full covariance matrix. The cell e.s.d.'s are taken into account individually in the estimation of e.s.d.'s in distances, angles and torsion angles; correlations between e.s.d.'s in cell parameters are only used when they are defined by crystal symmetry. An approximate (isotropic) treatment of cell e.s.d.'s is used for estimating e.s.d.'s involving l.s. planes.
Refinement. Refinement of *F*^2^ against ALL reflections. The weighted *R*-factor *wR* and goodness of fit *S* are based on *F*^2^, conventional *R*-factors *R* are based on *F*, with *F* set to zero for negative *F*^2^. The threshold expression of *F*^2^ > σ(*F*^2^) is used only for calculating *R*-factors(gt) *etc*. and is not relevant to the choice of reflections for refinement. *R*-factors based on *F*^2^ are statistically about twice as large as those based on *F*, and *R*- factors based on ALL data will be even larger.

### Fractional atomic coordinates and isotropic or equivalent isotropic displacement parameters (Å^2^)

**Table:**

	*x*	*y*	*z*	*U*_iso_*/*U*_eq_	Occ. (<1)
Fe1	1.0000	0.15100 (2)	0.2500	0.01928 (13)	
Cl1	0.85420 (5)	0.21251 (3)	0.17847 (11)	0.03440 (18)	
Cl2	0.99637 (5)	0.08774 (3)	0.00604 (10)	0.03183 (17)	
O1	1.0000	0.68189 (19)	0.2500	0.139 (3)	
H1	0.9766	0.6682	0.1350	0.208*	0.50
N1	0.89657 (16)	0.56531 (10)	0.1150 (3)	0.0181 (4)	
C1	0.7488 (2)	0.63346 (14)	−0.0768 (4)	0.0304 (6)	
H1A	0.7225	0.6522	0.0235	0.046*	
H1B	0.6883	0.6291	−0.1985	0.046*	
H1C	0.8039	0.6624	−0.0974	0.046*	
C2	0.79690 (19)	0.56745 (13)	−0.0131 (3)	0.0232 (5)	
C3	0.7407 (2)	0.50876 (14)	−0.0831 (4)	0.0290 (6)	
H3	0.6692	0.5103	−0.1729	0.035*	
C4	0.7888 (2)	0.44989 (14)	−0.0223 (4)	0.0305 (6)	
H4	0.7508	0.4105	−0.0709	0.037*	
C5	0.8949 (2)	0.44681 (12)	0.1129 (4)	0.0242 (5)	
C6	0.94610 (18)	0.50710 (11)	0.1799 (3)	0.0178 (4)	
C7	0.9500 (2)	0.38679 (12)	0.1836 (4)	0.0323 (6)	
H7	0.9157	0.3462	0.1363	0.039*	
C8	1.0000	0.7446 (2)	0.2500	0.073 (2)	
H8A	0.9305	0.7606	0.2564	0.088*	0.50
H8B	1.0107	0.7606	0.1307	0.088*	0.50
H8C	1.0587	0.7606	0.3629	0.088*	0.50
H1D	0.924 (9)	0.592 (5)	0.147 (16)	0.088*	0.50

### Atomic displacement parameters (Å^2^)

**Table:**

	*U*^11^	*U*^22^	*U*^33^	*U*^12^	*U*^13^	*U*^23^
Fe1	0.0158 (2)	0.0150 (2)	0.0223 (2)	0.000	−0.00025 (17)	0.000
Cl1	0.0220 (3)	0.0221 (3)	0.0523 (4)	0.0066 (2)	0.0028 (3)	0.0083 (3)
Cl2	0.0328 (3)	0.0324 (3)	0.0289 (3)	−0.0084 (3)	0.0083 (3)	−0.0095 (2)
O1	0.154 (5)	0.0212 (19)	0.141 (5)	0.000	−0.090 (5)	0.000
N1	0.0187 (9)	0.0185 (9)	0.0181 (9)	−0.0025 (7)	0.0074 (7)	−0.0004 (7)
C1	0.0194 (11)	0.0393 (15)	0.0290 (13)	0.0023 (10)	0.0030 (9)	0.0067 (11)
C2	0.0200 (11)	0.0314 (13)	0.0195 (11)	−0.0052 (9)	0.0085 (9)	−0.0008 (9)
C3	0.0216 (11)	0.0415 (15)	0.0254 (12)	−0.0124 (10)	0.0097 (10)	−0.0077 (11)
C4	0.0309 (13)	0.0343 (14)	0.0327 (14)	−0.0189 (11)	0.0192 (11)	−0.0154 (11)
C5	0.0307 (12)	0.0204 (11)	0.0297 (13)	−0.0092 (10)	0.0211 (10)	−0.0062 (9)
C6	0.0192 (10)	0.0181 (10)	0.0193 (10)	−0.0012 (8)	0.0107 (9)	−0.0012 (8)
C7	0.0450 (15)	0.0153 (11)	0.0507 (17)	−0.0049 (10)	0.0349 (14)	−0.0062 (10)
C8	0.099 (5)	0.026 (2)	0.070 (4)	0.000	−0.005 (4)	0.000

### Geometric parameters (Å, º)

**Table:**

Fe1—Cl2	2.1826 (8)	C2—C3	1.409 (4)
Fe1—Cl2^i^	2.1826 (8)	C3—C4	1.359 (4)
Fe1—Cl1^i^	2.1966 (8)	C3—H3	0.9500
Fe1—Cl1	2.1966 (8)	C4—C5	1.416 (4)
O1—C8	1.277 (6)	C4—H4	0.9500
O1—H1	0.8400	C5—C6	1.408 (3)
N1—C2	1.333 (3)	C5—C7	1.427 (4)
N1—C6	1.360 (3)	C6—C6^i^	1.445 (4)
N1—H1D	0.64 (11)	C7—C7^i^	1.349 (6)
C1—C2	1.493 (4)	C7—H7	0.9500
C1—H1A	0.9800	C8—H8A	0.9800
C1—H1B	0.9800	C8—H8B	0.9800
C1—H1C	0.9800	C8—H8C	0.9800
			
Cl2—Fe1—Cl2^i^	107.61 (5)	C4—C3—H3	120.0
Cl2—Fe1—Cl1^i^	108.48 (3)	C2—C3—H3	120.0
Cl2^i^—Fe1—Cl1^i^	110.92 (4)	C3—C4—C5	120.6 (2)
Cl2—Fe1—Cl1	110.92 (4)	C3—C4—H4	119.7
Cl2^i^—Fe1—Cl1	108.48 (3)	C5—C4—H4	119.7
Cl1^i^—Fe1—Cl1	110.41 (4)	C6—C5—C4	116.7 (2)
C8—O1—H1	109.5	C6—C5—C7	119.7 (2)
C2—N1—C6	121.2 (2)	C4—C5—C7	123.6 (2)
C2—N1—H1D	122 (10)	N1—C6—C5	121.5 (2)
C6—N1—H1D	117 (10)	N1—C6—C6^i^	119.29 (12)
C2—C1—H1A	109.5	C5—C6—C6^i^	119.25 (14)
C2—C1—H1B	109.5	C7^i^—C7—C5	121.03 (16)
H1A—C1—H1B	109.5	C7^i^—C7—H7	119.5
C2—C1—H1C	109.5	C5—C7—H7	119.5
H1A—C1—H1C	109.5	O1—C8—H8A	109.5
H1B—C1—H1C	109.5	O1—C8—H8B	109.5
N1—C2—C3	120.0 (2)	H8A—C8—H8B	109.5
N1—C2—C1	117.6 (2)	O1—C8—H8C	109.5
C3—C2—C1	122.3 (2)	H8A—C8—H8C	109.5
C4—C3—C2	120.0 (2)	H8B—C8—H8C	109.5
			
C6—N1—C2—C3	0.3 (3)	C2—N1—C6—C6^i^	−179.5 (3)
C6—N1—C2—C1	−179.8 (2)	C4—C5—C6—N1	−1.1 (3)
N1—C2—C3—C4	−1.0 (4)	C7—C5—C6—N1	179.5 (2)
C1—C2—C3—C4	179.1 (3)	C4—C5—C6—C6^i^	179.2 (3)
C2—C3—C4—C5	0.6 (4)	C7—C5—C6—C6^i^	−0.2 (4)
C3—C4—C5—C6	0.4 (4)	C6—C5—C7—C7^i^	1.1 (5)
C3—C4—C5—C7	179.7 (3)	C4—C5—C7—C7^i^	−178.2 (3)
C2—N1—C6—C5	0.8 (3)		

Symmetry code: (i) −*x*+2, *y*, −*z*+1/2.

### Hydrogen-bond geometry (Å, º)

**Table:**

*D*—H···*A*	*D*—H	H···*A*	*D*···*A*	*D*—H···*A*
O1—H1···N1	0.84	2.33	2.751 (4)	112
N1—H1*D*···O1	0.65 (12)	2.10 (10)	2.751 (4)	175 (14)
